# Reproducing Cold-Chain Conditions in Real Time Using a Controlled Peltier-Based Climate System

**DOI:** 10.3390/s25216689

**Published:** 2025-11-01

**Authors:** Javier M. Garrido-López, Alfonso P. Ramallo-González, Manuel Jiménez-Buendía, Ana Toledo-Moreo, Roque Torres-Sánchez

**Affiliations:** Department of Automation, Electrical Engineering and Electronic Technology, ETSII, Universidad Politécnica de Cartagena (UPCT), European University of Technology, 30202 Cartagena, Spain; javier.garrido@upct.es (J.M.G.-L.); alfonsop.ramallo@upct.es (A.P.R.-G.); ana.toledo@upct.es (A.T.-M.); roque.torres@upct.es (R.T.-S.)

**Keywords:** thermoelectric climate chamber, cold-chain simulation, cascade 2-DOF PID control, physical twin, shelf-life prediction, supply-chain optimization, environmental sensing

## Abstract

Temperature excursions during refrigerated transport strongly affect the quality and shelf life of perishable food, yet reproducing realistic, time-varying cold-chain temperature histories in the laboratory remains challenging. In this study, we present a compact, portable climate chamber driven by *Peltier* modules and an identification-guided control architecture designed to reproduce real refrigerated-truck temperature histories with high fidelity. Control is implemented as a cascaded regulator: an outer two-degree-of-freedom PID for air-temperature tracking and faster inner PID loops for module-face regulation, enhanced with derivative filtering, anti-windup back-calculation, a Smith predictor, and hysteresis-based bumpless switching to manage dead time and polarity reversals. The system integrates distributed temperature and humidity sensors to provide real-time feedback for precise thermal control, enabling accurate reproduction of cold-chain conditions. Validation comprised two independent 36-day reproductions of field traces and a focused 24-h comparison against traditional control baselines. Over the long trials, the chamber achieved very low long-run errors (MAE≅0.19 °C, MedAE≅0.10 °C, RMSE≅0.33 °C, $R^{2}=0.9985$). The 24-h test demonstrated that our optimized controller tracked the reference, improving both transient and steady-state behaviour. The system tolerated realistic humidity transients without loss of closed-loop performance. This portable platform functions as a reproducible physical twin for cold-chain experiments and a reliable data source for training predictive shelf-life and digital-twin models to reduce food waste.

## 1. Introduction

Perishable-food loss during storage and transport remains a pressing global problem [1,2,3]: temperature excursions and variable thermal histories incurred along the cold chain accelerate biochemical change and microbial growth, shortening shelf life and increasing waste [4,5,6]. Experimental and modeling efforts to predict and mitigate these losses depend on being able to reproduce, in a repeatable way, the complex, time-varying temperature trajectories that products experience in real logistics operations (e.g., loading/unloading, door openings, multi-modal transfers) [7,8,9].

Laboratory environmental chambers and conventional compressor-based refrigeration are excellent for maintaining steady setpoints, but they are limited when the objective is to emulate arbitrary, rapidly changing profiles [10,11,12]. In addition to their environmental impact, mechanical inertia, refrigerant dynamics, and compressor cycling restrict attainable bandwidth, and compressor systems are not well-suited to frequent polarity-reversible heating/cooling in compact, portable test rigs [10,12,13,14]. For research groups and industry partners who need realistic, repeatable emulation without large infrastructure, this limitation is important and motivates alternative actuator and control solutions.

Thermoelectric (Peltier) modules are a promising alternative for compact, reversible thermal actuation. They are solid-state, polarity-reversible, and capable of fine, fast electronic control, making them attractive for small-scale devices that must switch between heating and cooling or follow aggressive temperature trajectories [15,16]. At the same time, thermoelectrics have specific physical and control challenges: electro-thermal coupling (Peltier and Seebeck effects), Joule heating and conduction, significant thermal inertia of the module plus heatsinking assembly, and a coefficient of performance (COP) that depends strongly on the temperature difference across the module and on drive current [15,17]. These characteristics produce nonlinear, sometimes asymmetric dynamics between heating and cooling that complicate controller design and require care to avoid integrator windup, actuator chattering, and inefficient operation [15,18].

Prior control attempts for Peltier-based systems range from simple PI controllers to sliding-mode, fuzzy, adaptive, and model-predictive (MPC) schemes [19,20,21,22,23,24,25,26,27]. Sliding-mode and adaptive laws can be effective in constrained laboratory problems but often need extensive tuning and may be sensitive to unmodelled dynamics [28,29]. Fuzzy and MPC approaches can be computationally heavy or require accurate predictive models, which can be a practical barrier for embedded or low-cost deployments [27,28,30]. Meanwhile, naive PID (Proportional–Integral–Derivative) designs frequently suffer from windup and poor transient behaviour during polarity switching and large setpoint ramps [27,28,31].

PID control remains one of the most widely used strategies in industrial and embedded systems due to its simplicity, robustness, and ease of implementation [28]. In its basic form, it combines proportional (reducing present error), integral (eliminating steady-state offset), and derivative (providing predictive damping of future error) actions to shape closed-loop performance. However, classical PID controllers often struggle with systems that exhibit nonlinearities, dead time, actuator saturation, or asymmetric dynamics—such as thermoelectric modules used for reversible heating and cooling [31].

To address these challenges, advanced PID variants have been developed. Two-degree-of-freedom (2-DOF) architectures decouple setpoint tracking from disturbance rejection by introducing two tuning weights for the proportional and derivative terms. These architectures allow finer control over transient behaviour and overshoot [32,33,34]. A complementary practical improvement is derivative filtering, where the derivative term includes a low-pass filter to retain predictive damping without amplifying measurement noise from the sensors [34].

Integral windup due to actuator saturation is usually prevented by implementing anti-windup back-calculation, which dynamically reduces the integrator state when the actuator saturates [35]. Dead time further degrades PID performance if untreated. The Smith predictor is a classical, low-complexity compensation technique that uses an internal model of the plant to predict the non-delayed output [33,36].

Cascade control is often useful when the physical system has a clear separation of time scales [33], as is the case with thermoelectric modules, which respond significantly faster than the air mass inside a chamber. That is why this is the most appropriate option for our application at hand. All these enhancements are critical for maintaining stability and performance in systems with actuator saturation and transport delays. For the goal of producing repeatable physical-twin experiments—long, field-faithful thermal histories that can feed food shelf-life models—a controller must be robust, computationally lightweight, and specifically designed to handle the thermoelectric idiosyncrasies above.

In this work, we propose and validate a pragmatic hardware-software solution that addresses these needs. The system couples an array of off-the-shelf thermoelectric modules, heatsinking, and forced convection with distributed sensing and an embedded cascade control architecture. The control strategy integrates an outer 2-DOF PID for air-temperature tracking with faster inner PID loops for module-face regulation [32,33,34]. The design adds derivative filtering, anti-windup back-calculation, Smith predictor’s dead-time compensation, and hysteresis-based bumpless switching to manage heating/cooling polarity changes [33,35,36]. Rather than relying on heavy real-time optimization, this approach leverages identification-derived models to guide tuning, keeping implementation lightweight and robust.

Accurate reproduction of cold-chain conditions requires not only precise thermal actuation but also reliable sensing [19]. Sensors play a critical role in capturing the dynamic temperature and humidity profiles within the chamber, enabling real-time feedback for closed-loop control. Distributed temperature and humidity sensors (NTC thermistors for module-face measurements and digital sensors for inside air) ensure spatial coverage and measurement fidelity, which are essential for replicating realistic transport scenarios and for generating high-quality data to train predictive shelf-life models [7,8,19]. Without robust sensing, control accuracy and experimental reproducibility would be severely compromised.

Our study introduces a novel approach with the following key contributions: (i) development of a compact, portable thermoelectric climate chamber with a lumped thermal-electrical model that relates module-face inputs to chamber air temperature; (ii) formulation of a practical identification and control workflow—system identification, separate heating/cooling inner-loop tuning, and cascade 2-DOF PID design with dead-time compensation and anti-windup—that achieves reliable, repeatable tracking of aggressive real-world profiles while remaining suitable for embedded deployment; and (iii) extensive experimental validation: identification experiments, controller comparisons and long-duration reproductions of refrigerated-truck traces (two independent 36-day trials and a focused 24-h test) that demonstrate sub-degree tracking accuracy, robustness to humidity transients and clear improvements over classical control and tuning baselines.

## 2. Materials and Methods

### 2.1. Peltier Modules

The thermal regulation in the climate chamber built for this study relies on thermoelectric modules (or Peltier cells). These solid-state devices use the so-called Peltier effect to transport heat between their two faces when an electrical current passes through them. Reversing the current reverses the direction of heat flow; hence, a single module can provide either cooling or heating at the internal cavity simply by changing the current polarity [15].

**Figure 1:**
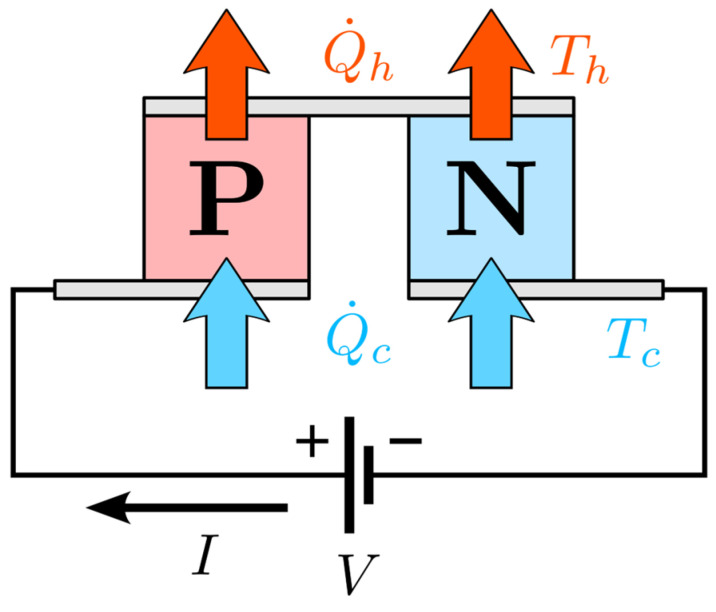
Diagram of a basic thermocouple pair in a Peltier module. Here, $T_{h}$: hot face temperature; $T_{c}$: cold face temperature; ${\dot{Q}}_{h}$: hot-side rejected heat; and ${\dot{Q}}_{c}$: cold-side absorbed heat.

A Peltier module is composed of many elemental thermocouples as basic units: paired P-type and N-type semiconductor legs electrically connected by metal interconnects and sandwiched between two ceramic plates that serve as thermal interfaces to the hot and cold sides. A diagram of one such thermocouple pair is provided in Figure 1. The elements are arranged electrically in series and thermally in parallel inside the module to obtain the required voltage/current and heat-pumping capacity.

#### 2.1.1. Fundamental Heat and Electrical Relations

The dominant physical effects at the device level are the Peltier heat flow, Joule heating, and thermal conduction between faces. When a current flows through the cell, heat is generated or absorbed at the junctions because charge carriers exchange energy while crossing material interfaces—this is the Peltier effect [37]. The heat flow associated with it is proportional to the current, as shown in (1) [17,37,38]:

(1) $${\dot{Q}}_{Peltier}=\Pi I=SIT$$

where ${\dot{Q}}_{Peltier}$ is the ideal Peltier heat, I is the current through the device, and Π=TS is the Peltier coefficient (with S the Seebeck coefficient and T the junction temperature).

The electrical power input to the module is $P_{e}=V_{e}I$, where the applied voltage $V_{e}$ must overcome the Seebeck voltage—produced by the temperature difference ΔT between faces—and must also drive current through the module resistance R, which produces Joule heating, as presented in (2) [15,39]:

(2) $$P_{e}=V_{e}I=S\Delta TI+RI^{2}$$

where $\Delta T=T_{h}-T_{c}$, with $T_{h}$ and $T_{c}$ referring to the hot and cold face temperatures, respectively. Accounting for all dominant heat terms yields the standard cold-side and hot-side heat balances used for module-level design. The cold-side heat absorbed by the module (${\dot{Q}}_{c}$) in absolute value equals the Peltier cooling at the cold junction minus half the Joule heating and minus conductive leakage from hot to cold through the module, as depicted in (3) [15,17,40]:

(3) $${\dot{Q}}_{c}=SIT_{c}-\frac{1}{2}I^{2}R-K\Delta T$$

where K is the thermal conductance. Energy conservation then gives the hot-side rejected heat (${\dot{Q}}_{h}$), as shown in (4), as the sum of the electrical power and the absorbed heat [17,40]:

(4) $${\dot{Q}}_{h}=P_{e}+{\dot{Q}}_{c}=SIT_{h}+\frac{1}{2}I^{2}R-K\Delta T$$

A commonly used performance metric is the coefficient of performance (COP), defined here for the cold and hot outputs as presented in (5) [15,17]:

(5) $$COP_{c}=\frac{{\dot{Q}}_{c}}{P_{e}}\;\;\;\;\;\;\;\;\;\;\;COP_{h}=\frac{{\dot{Q}}_{h}}{P_{e}}=1+COP_{c}$$

#### 2.1.2. Parameter Extraction and Effective Lumped Quantities

Module effective electrical and thermal parameters can be related to geometry and material properties as given in Equations (6)–(8) [41]:

(6) $$R=\frac{2N_{u}\rho_{e}}{G}$$

(7) $$K=2N_{u}k_{\theta }G$$

(8) $$S=2N_{u}s_{\alpha }$$

where $N_{u}$ denotes the number of basic thermocouple pairs in a module, $\rho_{e}$ the electrical resistivity (Ωm), G the geometrical factor (area/length, m), $k_{\theta }$ the thermal conductivity ($Wm^{-1}K^{-1}$) and $s_{\alpha }$ the elemental Seebeck coefficient ($VK^{-1}$). Alternatively, these parameters can be inferred directly from datasheet performance points ($\Delta T_{max}$, $I_{max}$, $V_{max}$), as depicted in Equations (9)–(11) [42]:

(9) $$R=\frac{\left(T_{h0}-\Delta T_{max}\right)V_{max}}{T_{h0}I_{max}}$$

(10) $$K=\frac{\left(T_{h0}-\Delta T_{max}\right)V_{max}I_{max}}{2T_{h0}\Delta T_{max}}$$

(11) $$S=\frac{V_{max}}{T_{h0}}$$

where $T_{h0}$ is the reference hot-side temperature used in the datasheet entry. For this project, we selected TEC1-12708 modules from HB Electronic Components (Pudong Avenue, 1139, Shanghai, China) [43]. The extracted parameters are summarized in Table 1.

#### 2.1.3. Performance Trends and Practical Implications

**Figure 2:**
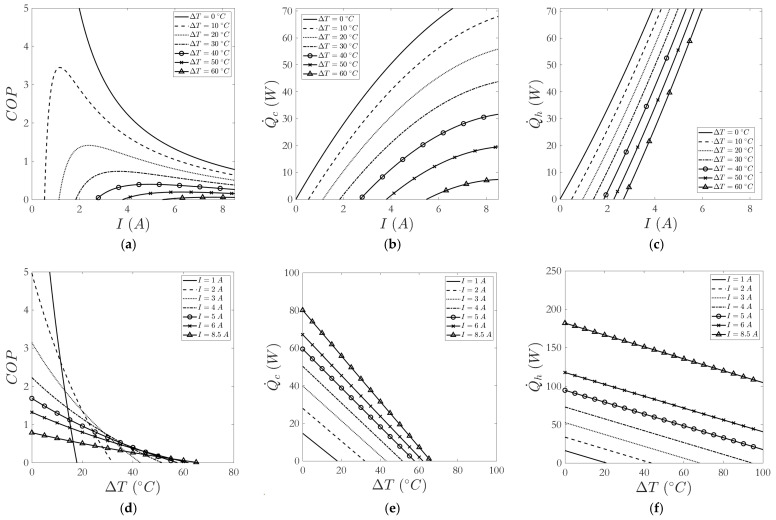
Representative Peltier cell curves. (**a**) $COP_{c}$, (**b**) ${\dot{Q}}_{c}$ and (**c**) ${\dot{Q}}_{h}$ versus current for various ΔT; (**d**) $COP_{c}$, (**e**) ${\dot{Q}}_{c}$ and (**f**) ${\dot{Q}}_{h}$ versus ΔT for various currents.

Figure 2 displays the calculated $COP_{c}$, ${\dot{Q}}_{c}$ and ${\dot{Q}}_{h}$ curves as functions of current and ΔT, obtained by evaluating Equations (2)–(5) with the module parameters:

Based on these curves, the following key insights were derived, which guided the climate chamber design and drove controller and thermal-management choices:

(i) Figure 2a shows interesting information about the performance of Peltier cells. The COP of the cell decreases as its current consumption—i.e., electrical power—increases, and this parameter is also low for very low consumptions. However, it presents an optimum value when the consumption of the cell is approximately 15–20% of its nominal value, which allows the highest amount of heat to be extracted with the lowest electrical consumption. Furthermore, the performance is higher the smaller the temperature difference between the cell faces.
(ii) In Figure 2b, we observe that a higher power consumption of the cell allows a greater amount of heat to be absorbed, although this increase shows asymptotic trends as the consumption increases. Thus, as ΔT decreases, larger increases in ${\dot{Q}}_{c}$ are observed when the consumption is low, up to approximately 50% of the nominal consumption.
(iii) Similarly, in Figure 2c, an increase in heat rejected is observed as consumption increases. However, in this case, the growth is exponential, which causes the fact that, for the same consumption, a lower ΔT implies a large increase in ${\dot{Q}}_{h}$.
(iv) Another interesting result is shown in Figure 2d. The performance of the cells is higher the lower the power consumption, and it is increasing as ΔT is lower, with a hyperbolic shape. However, this trend is true until 40–50% of the maximum ΔT allowed by the cell is reached, at which point the curves cross and a higher current consumption allows a higher COP for the same ΔT. This also implies that, for a given ΔT, at the cutoff points of the curves, the same COP can be reached for different current values. In these cases, if a fast dynamic is sought, it could be interesting to supply the cell with the higher current value, since it would achieve a higher heat extraction (Figure 2b), while maintaining the same efficiency. If reducing consumption while maintaining that ΔT is required, it may be of more interest to select the lower current.
(v) Finally, Figure 2e,f show that the evolution of ${\dot{Q}}_{c}$ and ${\dot{Q}}_{h}$ upon variation of ΔT is very linear, with both values growing as current consumption increases. In the case of ${\dot{Q}}_{h}$, there is always an increase as the power consumption rises, as can be clearly seen in the curve with the highest consumption (8.5 A), which increases its value of ${\dot{Q}}_{h}$ quite a lot for the same ΔT. On the contrary, for ${\dot{Q}}_{c}$, with the same consumption of 8.5 A, there is no significant difference with respect to the previous consumption value (6 A), as it tends to stabilize. This is because ${\dot{Q}}_{h}={\dot{Q}}_{c}+P_{e}$ and, while $P_{e}$ is increasing, ${\dot{Q}}_{c}$ does not vary much in value at high consumptions.

### 2.2. Design and Construction of a Peltier-Based Climate Chamber

**Figure 3:**
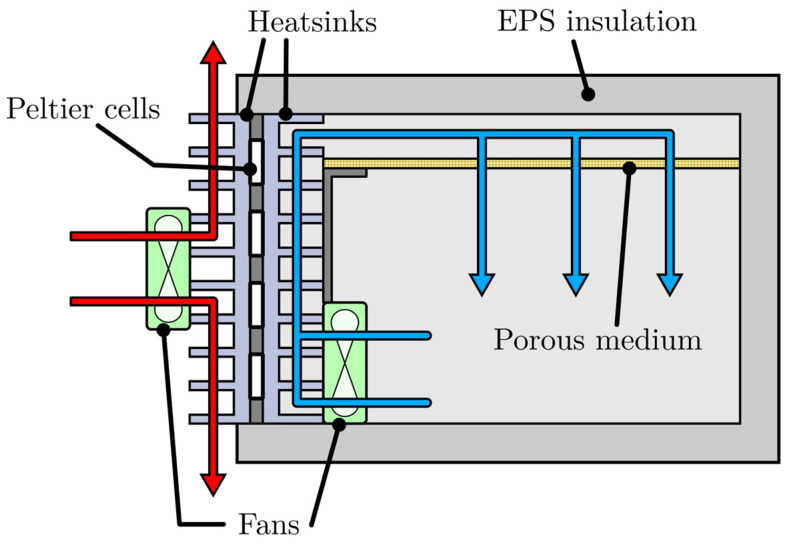
System diagram of the designed climate chamber.

**Figure 4:**
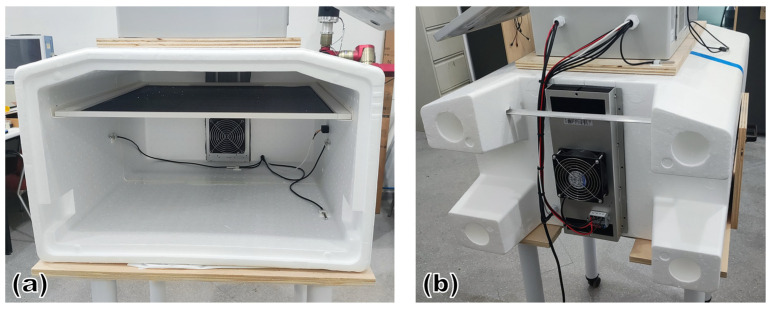
Prototype of the climate chamber: (**a**) detail of the interior and the porous medium; (**b**) detail of the heat exchangers with the fans.

Figure 3 shows a system diagram of the designed climate chamber (the figure illustrates cooling mode; reversing the current would provide heating). Figure 4 presents the prototype of the device. The chamber consisted of a rectangular insulated container with internal dimensions 600×600×400 mm and 50 mm thick walls. It was constructed from expanded polystyrene (EPS).

#### 2.2.1. Mounting of the Thermoelectric Modules and Heatsinking

Eight Peltier modules, with their faces in contact with two aluminum heatsinks (400×150 mm, 30 mm fins), were installed in a hole at the rear of the chamber. One heatsink faced the internal cavity, and the other faced the ambient. Forced convection was provided by 11 W DC fans mounted on both heatsinks. The external fan maintained the outer face of the cells near room temperature, while the internal fan distributed conditioned air inside.

Operating at low temperatures could produce condensate on cold surfaces, notably on the heatsinks. For that, a drainage system was included to discharge the water generated by air condensation from the interior.

#### 2.2.2. Air Distribution and Homogenization

To reduce spatial temperature non-uniformities, we combined directed forced convection with a passive flow-conditioning element placed near the chamber ceiling. A fibreglass mesh framed by aluminum profiles acted as a porous medium upstream of the conditioned volume.

The purpose of this element was to: (a) attenuate inlet jets and local recirculation; (b) produce a smoother downstream velocity field and more uniform temperature distribution; and (c) counteract stratification caused by natural convection (during heating, it helps move warm air downward; during cooling, it forces cold air to descend rather than pool at the floor). The internal fan blows upward from the side, and conditioned air then descends through this diffuser.

### 2.3. Integration of Electronics, Sensors, and Instrumentation

The ESP32 System-On-Chip from Espressif Systems (Bibo Road, 690, Shanghai, China) [44] was used as the chamber’s central controller. It was selected for its high performance, low cost, and versatility, and for incorporating a 12-bit analogue-to-digital converter (ADC), built-in peripherals for communications, and local data storage (8 MB flash memory) for storing timestamped experimental logs.

**Figure 5:**
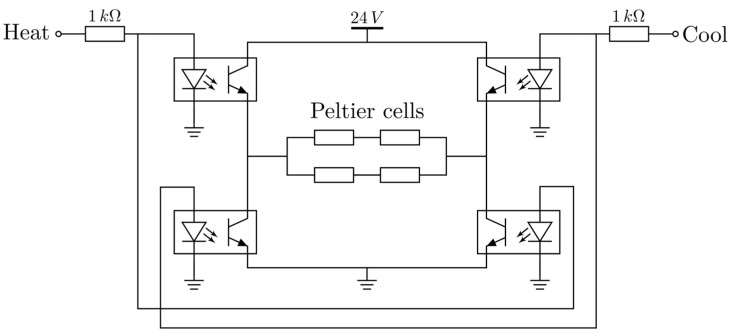
H-bridge to control Peltier cells heating and cooling.

Bidirectional control of the thermoelectric modules was achieved with a custom H-bridge that incorporated four optocouplers to electrically isolate the logic outputs from the power stage (Figure 5). The H-bridge was driven by a 1 kHz PWM (Pulse Width Modulation) signal from the ESP32 to modulate the effective power applied to the modules.

The cells were grouped electrically in pairs—two cells in series, and groups connected in parallel—and supplied from a 24 V DC bus, such that each cell operated nominally at 12 V. The H-bridge allowed controlling cell polarity, for heating or cooling. The system included snubbing circuits to protect switching elements from transient peak values and overcurrent protection for safety and to prevent module overheating.

The sensors integrated into the climate chamber were essential for enabling accurate, real-time thermal regulation. Temperature measurements were obtained from NTC (Negative Temperature Coefficient) thermistors (±0.5 °C) placed on representative hot and cold faces of the Peltier modules, providing fast local feedback for the inner control loop. Chamber air temperature and relative humidity were monitored using SHTC3 (±0.2 °C, ±2% RH)—from Sensirion (Laubisruetistrasse, 50, Stäfa, Switzerland) [45]—and DS18B20 (±0.5 °C)—from Dallas Semiconductor (South Beltwood Parkway, 4401, Dallas, TX, USA) [46]—sensors, which were spatially distributed to capture gradients and ensure uniformity across the conditioned volume.

These sensors and instrumentation served multiple roles: (i) they provided fast, reliable feedback required for closed-loop PID control, (ii) they enabled system identification and model calibration, and (iii) they ensured that the chamber accurately reproduced the dynamic temperature and humidity profiles observed in real refrigerated transport. Sensor data were sampled and logged with timestamps via the ESP32 microcontroller, using its 12-bit ADC operating at a 100 kHz sampling rate. This setup ensured high-resolution data acquisition suitable for post-experiment analysis and full traceability.

Without precise and responsive sensing, the control system would be unable to track aggressive setpoint changes or maintain fidelity during transients. Moreover, the quality of the thermal data generated by the chamber—used to train predictive shelf-life models—depends directly on the accuracy and reliability of the sensor measurements. Thus, sensing is not only a support function but a central component of the system’s ability to reduce perishable food waste through reproducible cold-chain emulation.

**Figure 6:**
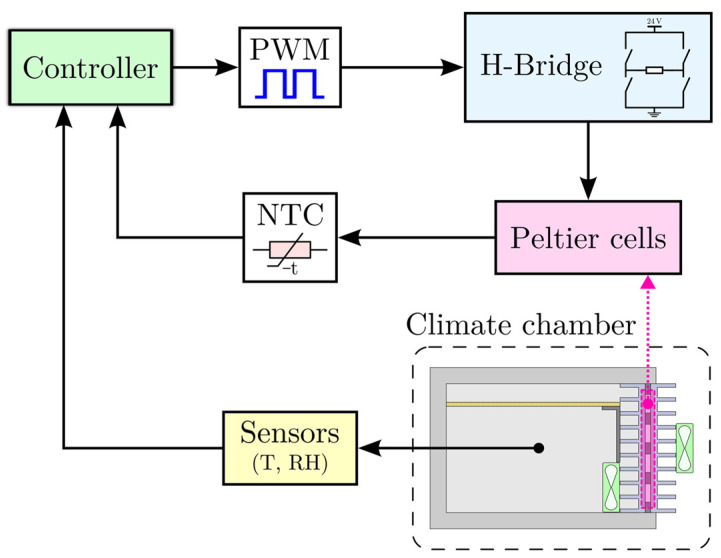
Full diagram of the measurement and control setup.

Sensors were housed in 3D-printed PLA (polylactic acid) enclosures to protect them from condensing moisture while permitting adequate airflow across sensing elements. All the electronics were placed inside an ABS electrical panel with IP65 protection. The complete diagram of the measurement and control system is shown in Figure 6.

The sensor subsystem was not only responsible for monitoring environmental conditions but also played a central role in the control methodology. The temperature and humidity data acquired in real time were used both for feedback in the control loops and for offline system identification and model validation. This integration ensured that the chamber could accurately reproduce dynamic cold-chain conditions and generate high-quality datasets for predictive modelling.

### 2.4. Dynamic Modelling of the Thermal Behaviour of the System

To enable model-based control design and implement the control system, we developed a thermal dynamic model of the chamber and the thermoelectric modules based on energy balance principles. The chamber interior was modelled as a single, well-mixed thermal control volume—a common assumption in climate chamber modelling due to its balance between accuracy and tractability—whose temperature, $T_{in}\left(t\right)$, evolved according to an energy balance. This accounted for the heat delivered from the modules through convection and heat losses through the enclosure, as presented in (12):

(12) $$\rho VC_{p}\frac{dT_{in}\left(t\right)}{dt}=\frac{T_{cell}\left(t\right)-T_{in}\left(t\right)}{R_{\theta }}-AU\left[T_{in}\left(t\right)-T_{\infty }\right]$$

where ρ is air density, V the internal volume, $C_{p}$ the specific heat of air, A the internal surface area of the chamber, U the overall heat-transfer coefficient of the enclosure, $T_{\infty }$ the external ambient temperature, $R_{\theta }$ the effective convection thermal resistance between the cells and the chamber air—including natural and forced convection—and $T_{cell}\left(t\right)$ the temperature of the modules. Thermal resistances between cells and heatsinks were neglected. The module thermal dynamics were described by the thermal equation depicted in (13):

(13) $$N_{c}m_{c}C_{p_{c}}\frac{dT_{cell}\left(t\right)}{dt}=N_{c}{\dot{q}}_{cell}\left(t\right)-\frac{T_{cell}\left(t\right)-T_{in}\left(t\right)}{R_{\theta }}$$

where $N_{c}=8$ is the number of cells, $m_{c}$ and $C_{p_{c}}$ are the lumped mass and specific heat of the module assembly (ceramic plates plus heatsink), and ${\dot{q}}_{cell}\left(t\right)$ is the heat produced (or absorbed) by a single module (signed positive for heating). Following the thermoelectric relations introduced in Section 2.1, the heat rate produced by a module is shown in (14):

(14) $${\dot{q}}_{cell}\left(t\right)=\pm SI_{cell}\left(t\right)T_{cell}\left(t\right)+\frac{1}{2}RI_{cell}{\left(t\right)}^{2}-K\left[T_{cell}\left(t\right)-T_{\infty }\right]$$

where $I_{cell}\left(t\right)$ is the current through the cell. The upper sign applies during heating and the lower sign during cooling (we adopt the convention that ${\dot{q}}_{cell}>0$ for heating and ${\dot{q}}_{cell}<0$ for cooling). The module’s electrical current depends on the applied module voltage $V_{cell}\left(t\right)$ and the Seebeck voltage, as given in (15):

(15) $$I_{cell}\left(t\right)=\frac{V_{cell}\left(t\right)\mp S\left[T_{cell}\left(t\right)-T_{\infty }\right]}{R}$$

Finally, the modules were driven through a PWM-controlled H-bridge. For a cell supply rail $V_{CC}$ and a duty cycle $D\left(t\right)\in \left[0,\;1\right]$ (where polarity was handled by the H-bridge logic), the voltage seen by each module was calculated as shown in (16):

(16) $$V_{cell}\left(t\right)=V_{CC}D\left(t\right)$$

#### Linearization and Laplace-Domain Modelling

Due to the nonlinear nature of the dynamic equations, it was necessary to perform a linearization process around operating points to work more easily in the Laplace domain. Given a nonlinear equation $F\left(x\right)=0$, where $x=(x_{1},x_{2},\ldots ,\;x_{n})$ are its n time-dependent variables—including the derivatives—and given an equilibrium point $x_{0}=\left(x_{1_{0}},\;x_{2_{0}},\ldots ,\;x_{n_{0}}\right)$—where $F\left(x_{0}\right)=0$ and all derivatives vanish—first order Taylor was applied as indicated in (17), which represents the linearized equation [47,48,49]:

(17) $$F\left(x\right)≅F\left(x_{0}\right)+{\left.\frac{\partial F}{\partial x_{1}}\right|}_{x_{0}}\Delta x_{1}+\cdots +{\left.\frac{\partial F}{\partial x_{n}}\right|}_{x_{0}}\Delta x_{n}=\sum_{i=1}^{n}{\left.\frac{\partial F}{\partial x_{i}}\right|}_{x_{0}}\Delta x_{i}=0$$

where $\Delta x_{i}=x_{i}-x_{i_{0}}$. Taking the Laplace transform yields the relation shown in (18), which was finally used for the control system:

(18) $$L\left\{F\left(x\right)\right\}≅\sum_{i=1}^{n}{\left.\frac{\partial F}{\partial x_{i}}\right|}_{x_{0}}L\left\{\Delta x_{i}\right\}=0$$

where $L\left\{f\left(t\right)\right\}=f\left(s\right)$ represents the transform of $f\left(t\right)$. From linearizing Equation (12) around $x_{01}=\left(T_{in_{0}},\;T_{cell_{0}}\right)$, the transfer function $G_{T}\left(s\right)$, which relates internal temperature $\Delta T_{in}(s)$ to cell temperature $\Delta T_{cell}\left(s\right)$, is displayed in (19). A transport delay L was included to model finite heat-transport and sensor/actuator dynamics:

(19) $$G_{T}\left(s\right)=\frac{\Delta T_{in}\left(s\right)}{\Delta T_{cell}\left(s\right)}=\frac{\Gamma }{\tau s+1}e^{-Ls}$$

where $\Gamma =c_{1}/\gamma$, $\tau =c_{2}\rho VC_{p}R_{\theta }/\gamma$, with $\gamma =1+R_{\theta }AU$, and $c_{1}$, $c_{2}$ are constants to be obtained experimentally. This relation was well-described by a first-order, time-delayed system. Similarly, combining Equations (13)–(16) and linearizing around $x_{02}=\left(T_{cell_{0}},I_{cell_{0}},\;D_{0}\right)$, the resulting transfer function had the form $G_{D}\left(s\right)=\frac{\Delta T_{cell}\left(s\right)}{\Delta D\left(s\right)}=K_{D}\frac{\tau_{z}s+1}{\left(\tau_{p_{1}}s+1\right)\left(\tau_{p_{2}}s+1\right)}$. When calculating the values approximately, $\tau_{z}\approx \tau_{p_{1}}$ was obtained. Therefore, the system could be approximated by (20) to relate $\Delta T_{cell}\left(s\right)$ with the duty cycle $\Delta D\left(s\right)$. Additionaly, considering a delay $L^{\prime }$ as follows:

(20) $$G_{D}\left(s\right)=\frac{\Delta T_{cell}\left(s\right)}{\Delta D\left(s\right)}≅\frac{\Omega }{\tau^{\prime }s+1}e^{-L^{\prime }s}$$

where $K_{D}=\pm \gamma N_{c}\frac{V_{CC}}{R}\left(ST_{cell_{0}}\pm RI_{cell_{0}}\right)/\left(AU+\delta \gamma \right)$, $\tau_{z}=\rho VC_{p}R_{\theta }/\gamma$, $\tau_{p_{1}}=2/(\alpha -\sqrt{\alpha^{2}-4\beta })$, $\tau_{p_{2}}=2/(\alpha +\sqrt{\alpha^{2}-4\beta })$, with $\delta =N_{c}\left[ST_{cell_{0}}\pm I_{cell_{0}}\left(R-S\right)+K\right]$, $\alpha =(1/R_{\theta }+\delta )/(N_{c}m_{c}C_{p_{c}})+\gamma /(\rho VC_{p}R_{\theta })$, $\beta =[AU/R_{\theta }+\delta (1/R_{\theta }+AU)]/(\rho VC_{p}N_{c}m_{c}C_{p_{c}})$, and with $\Omega =c_{3}K_{D}$, $\tau^{\prime }=c_{4}\tau_{p_{2}}$. Constants $c_{3}$ and $c_{4}$ must be determined experimentally. The delay $L^{\prime }$ differs from L and is different for heating and cooling. Also, it is a first-order plus time delay system where Ω and $\tau^{\prime }$ depend on cell heating or cooling, obtaining two different models.

The values of the operating points are given in Table 2, after evaluating the equations at that point. The equilibrium was selected at the midpoint of the system’s operating range. For clarity, we used D>0 for heating and D<0 for cooling, expressed as a percentage.

### 2.5. Control System Design for Thermal Management

**Figure 7:**
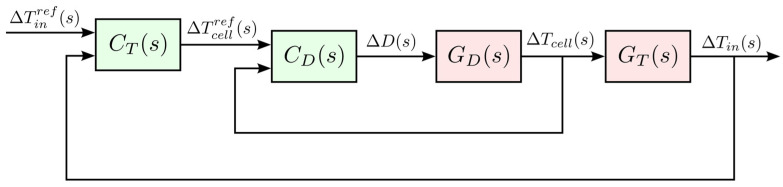
Block diagram of the cascade control architecture.

The chamber was regulated by a two-layer cascade control architecture. The outer (primary) loop commanded the chamber air temperature, and the inner (secondary) loop commanded the thermoelectric module temperature. This cascade architecture improved disturbance rejection and allowed the faster inner loop to stabilize module-level dynamics while the outer loop handled the slower air-temperature dynamics. Figure 7 illustrates the architecture block diagram.

#### 2.5.1. Controller Architecture

Both loops use a two-degree-of-freedom PID controller (2-DOF PID) extended with:

(i) A derivative filter to avoid amplifying measurement noise,
(ii) An anti-windup mechanism based on back-calculation to handle actuator saturation, and
(iii) A Smith predictor (with a low-pass filter) to compensate for the transport dead-time present in the thermal paths.

The 2-DOF PID control law implemented in the Laplace domain is presented in (21):

(21) $$U\left(s\right)=K_{p}\left[\left(\lambda R\left(s\right)-Y\left(s\right)\right)+\frac{1}{T_{i}s}\left(R\left(s\right)-Y\left(s\right)\right)+\frac{T_{d}s}{\eta T_{d}s+1}\left(\mu R\left(s\right)-Y\left(s\right)\right)\right]$$

where $R\left(s\right)$ is the reference, $Y\left(s\right)$ the measured output, $K_{p}$ the proportional gain, $T_{i}$ the integral time, $T_{d}$ the derivative time, η the derivative filter constant, and $\lambda ,\mu \in \left[0,\;1\right]$ the two-degree weighting factors that tune how reference changes influence the proportional and derivative terms, respectively. Choosing λ<1 or μ<1 reduces the immediate impact of setpoint steps on the proportional or derivative action and helps reduce overshoot for aggressive setpoint profiles [32,34,50].

#### 2.5.2. Anti-Windup Method

Integral windup was prevented using back-calculation. When the computed control signal u exceeds the actuator limits (clipped to $u_{sat}$), the integral error accumulator $e_{i}$ is adjusted to drive the integrator state toward the clipped value with a gain constant $K_{b}$, such that, if $u>u_{sat}$ then $e_{i}\leftarrow e_{i}-K_{b}\left(u-u_{sat}\right)$ [35].

This back-calculation removes accumulated integral action proportional to the current saturation amount and the tuning constant $K_{b}$, restoring the integrator as soon as the actuator leaves saturation. Actuator limits were enforced to protect the modules and power electronics. In our implementation, the practical saturation bounds were $\left[-15,\;165\right]\;{}^{\circ}C$ for module-face temperature and $\left[0,\;100\right]\%$ for duty cycle.

#### 2.5.3. Smith Predictor for Dead-Time Compensation

Thermal transport and sensing introduce non-negligible dead time in the plant. To reduce the negative effects of delay on closed-loop performance, we implemented a Smith predictor that uses a model of the non-delayed plant $\tilde{G}\left(s\right)$ and the delay θ. The filtered predictor output $\hat{Y}\left(s\right)$ was computed as shown in (22):

(22) $$\hat{Y}\left(s\right)=F_{\nu }\left(s\right)\left[Y\left(s\right)-\tilde{G}\left(s\right)e^{-\theta s}U\left(s\right)\right]+\tilde{G}\left(s\right)U\left(s\right)$$

This was applied with $F_{\nu }\left(s\right)=1/(\nu \theta s+1)$, a low-pass filter used to increase robustness to model mismatch and measurement noise [33,36]. The predictor output $\hat{Y}\left(s\right)$ was used inside the control law in place of the delayed plant output to reduce the effective loop delay seen by the controller.

#### 2.5.4. Cascade Implementation and Hysteresis-Based Switching Logic

The cascade control was implemented as follows:

- Outer loop $C_{T}\left(s\right)$ tracked the chamber air temperature increment $\Delta T_{in}^{ref}\left(s\right)$, and its output was a module temperature reference $\Delta T_{cell}^{ref}\left(s\right)$ that the inner loop had to achieve.
- Inner loop $C_{D}\left(s\right)$ received $\Delta T_{cell}^{ref}\left(s\right)$ and output the required duty-cycle increment $\Delta D\left(s\right)$ to the H-bridge driver.

Both controllers incorporated the 2-DOF PID, Smith predictor, and anti-windup. As the thermoelectric behaviour and time constants differ between heating and cooling—sign changes in the electro-thermal equations change gains and delays—two separate inner controllers were tuned: $C_{D}^{h}\left(s\right)$ for heating and $C_{D}^{c}\left(s\right)$ for cooling. The control system switched between these two PID laws according to the sign of the requested action (heating or cooling).

To prevent chattering when the desired action crossed zero, a hysteresis band of 0.5 °C was applied before switching branches. This value was chosen as a compromise between responsiveness and robustness: it was small enough to maintain accurate temperature control, yet sufficiently large to suppress unwanted switching due to measurement noise or small fluctuations near the setpoint. This ensured smooth transitions and reduced the probability of frequent toggling under noisy measurements.

To avoid abrupt control transients at the switch, the implementation also performed a bumpless transfer strategy: when switching, the integrator states and filtered derivative states were re-initialized to ensure that the command to the actuator did not suffer a step discontinuity. All these control features ensured stable, accurate reproduction of temperature trajectories while protecting hardware and minimizing transient overshoot.

### 2.6. Tuning and Optimization Strategies for the Controllers

Controller tuning followed a two-stage strategy: (i) obtain initial parameter sets from several established model-based tuning rules; and (ii) refine those sets experimentally on the real plant to balance tracking, robustness, and actuator effort.

#### 2.6.1. Initial Tuning from Model-Based Methods

For the system transfer functions (Section Linearization and Laplace-Domain Modelling), described by first-order plus dead-time models of the form $G\left(s\right)=\frac{\kappa }{Ts+1}e^{-\theta s}$, we computed candidate PID parameters ($K_{p}$, $T_{i}$, $T_{d}$) for both the outer air-temperature controller $C_{T}\left(s\right)$ and the inner controller $C_{D}\left(s\right)$ using the following tuning methods: Ziegler–Nichols (ZN), Cohen–Coon (CC), Chien–Hrones–Reswick (CHR), Approximate M-constrained Integral Gain Optimization (AMIGO), and Skogestad Internal Model Control (SIMC) (see Table 3) [51,52,53,54,55].

These tuning methods were selected because they are widely used in industrial PID applications and provide a range of trade-offs between responsiveness, robustness, and overshoot. Their performance was evaluated using standard control metrics to ensure objective comparison.

In addition, we evaluated the Multiple Dominant Pole Method (MDPM) for the 2-DOF PID configuration, where parameters are obtained from the dominant pole $s_{*}$ (see Table 4) [56]. These candidate tunings provided starting points that respected the dynamics and delays of the heating and cooling branches.

#### 2.6.2. Initial 2-DOF and Auxiliary Parameter Choices

Each loop used a two-degree-of-freedom PID, such that setpoint transients and disturbance rejection could be traded independently. The derivative term included a first-order filter to limit noise amplification. Initial settings for the 2-DOF weights and filters were: (a) proportional setpoint weighting: λ=0.7; (b) derivative setpoint weighting: μ=0; and (c) derivative filter: η=0.05 [57,58].

Anti-windup back-calculation was implemented with an initial back-calculation constant $K_{b}$ taken as $1/\sqrt{T}$ and $1/\sqrt{T_{i}T_{d}}$, while the Smith predictor’s low-pass filter was initialized with ν=0.5 [35,57,59]. These empirical choices provide stable starting behaviour for most first-order plants with moderate dead time.

#### 2.6.3. Performance Evaluation and Selection

To compare candidate tunings objectively, we simulated each controller on a representative test reference and computed four performance indicators: Integral Square Error (ISE), Integral Absolute Error (IAE), Integral of Time-multiplied Absolute Error (ITAE), and Total Variation (TV), according to Equations (23)–(26) [60,61]:

(23) $$\mathrm{ISE}=\int_{0}^{t_{s}}e^{2}\left(t\right)dt$$

(24) $$\mathrm{IAE}=\int_{0}^{t_{s}}\left|e\left(t\right)\right|dt$$

(25) $$\mathrm{ITAE}=\int_{0}^{t_{s}}t\left|e\left(t\right)\right|dt$$

(26) $$\mathrm{TV}=\int_{0}^{t_{s}}\left|\frac{du\left(t\right)}{dt}\right|dt$$

where $e\left(t\right)$ is the tracking error, $u\left(t\right)$ the control signal, and $t_{s}$ the settling time. The candidate tunings were evaluated for two demanding step tests that stress different parts of the operating range: a heating step of $\Delta T_{in}=+40\;{}^{\circ}C$ and a cooling step of $\Delta T_{in}=-10\;{}^{\circ}C$, each applied for 2 h. TV was used to quantify controller effort (actuator activity) and ISE, IAE, ITAE to quantify tracking quality and settling behaviour.

#### 2.6.4. Controller Fine Tuning

The best-performing candidates from the simulation comparison served as initial control settings. Fine-tuning was carried out iteratively on the physical chamber. The following procedure was used:

(1) Apply a step or short profile and observe the closed-loop response.
(2) Adjust $K_{p}$ to trade steady-state speed versus overshoot.
(3) Adjust $T_{i}$ to remove steady-state error while avoiding slow oscillatory behaviour.
(4) Tune $T_{d}$ (and η) to improve damping and reduce overshoot; reduce derivative action if noise amplification is observed.
(5) Adjust 2-DOF weights λ and μ if setpoint transitions produce excessive overshoot.
(6) Tune anti-windup constant $K_{b}$ to obtain fast recovery from actuator saturation without destabilizing the integrator.
(7) Tune the Smith predictor’s filter ν to improve robustness against model mismatch (longer ν increases robustness but reduces dead-time compensation effectiveness).

Final tuning aimed to minimize error indicators to ensure robust control, accuracy, no sustained oscillations, and safe actuator saturation behaviour. The final tuned controller parameters are reported in Section 3.2.

## 3. Results and Discussion

### 3.1. System-Identification Results

System identification confirmed that both the air-temperature loop and the module electro-thermal loop were well-approximated by first-order dynamics with transport delay, consistent with the model structure adopted in Section Linearization and Laplace-Domain Modelling.

The unknown constants in (16) and (17) (Γ, τ, L, Ω, $\tau^{\prime }$, $L^{\prime }$) were obtained experimentally by open-loop identification tests: (a) first, the chamber was brought to a well-defined steady operating point, allowing sufficient time for thermal transients to decay before applying excitation; (b) then a step input of amplitude Λ was applied in duty cycle switching cell polarity; (c) the first-order systems with the form $G\left(s\right)=\kappa /(Ts+1)$ were fitted to a curve $k\Lambda \left(1-e^{-t/T}\right)$ to estimate gains and time constants, ignoring time delay for the initial fit; and (d) the dead time was then determined graphically as the lag between inputs and outputs [47].

**Figure 8:**
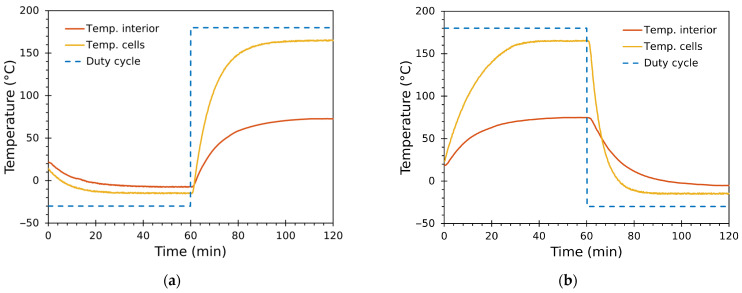
Representative step responses used for system identification: (**a**) heating (cool → heat); (**b**) cooling (heat → cool). Duty cycle was driven through its full range (±100%).

Figure 8 shows representative step responses used for identification. Figure 8a corresponds to the heating branch—cells driven from cool (D=−100%) to heat (D=100%)—and Figure 8b to the cooling branch—cells driven from heat (D=100%) to cool (D=−100%). In all tests, the duty cycle was driven through its full available range (±100%), and the module thermistors and distributed air sensors were recorded until steady state. The identified parameters for both plants are summarized in Table 5.

#### Interpretation and Implications from Identification Tests

The following key observations were derived from the identified parameters:

- Air-loop dynamics $G_{T}(s)$. The transfer from module face temperature to air temperature is slow: the dominant time constant is τ=26.85 min and the transport delay is L=1.17 min. The steady-state gain Γ=0.5441 indicates that approximately 0.54 °C of chamber air change is produced per 1 °C change at the module face, reflecting the moderate thermal mass and convective coupling of the chamber.
- Module dynamics $G_{D}(s)$. The mapping from PWM duty-cycle to module face temperature is significantly faster than the air loop, as required for cascade control. Identified time constants were $\tau_{heat}^{\prime }=8.84\;\min$ and $\tau_{cool}^{\prime }=18.17\;\min$; the shorter heating time constant indicates that the module assembly responds faster when driven to heat—likely due to different convective conditions and thermoelectric asymmetry. Transport delays $L^{\prime }$ were small (~0.8–1.0 min).
- Asymmetry between heating and cooling. The identified gains Ω differ markedly: $\Omega_{heat}=144.93$ versus $\Omega_{cool}=34.67$ (°C per unit duty-step). This large disparity reflects the inherently asymmetric electro-thermal behaviour of the modules and the mounting/heatsink arrangement. In practice, it requires separate inner-loop tuning for heating and cooling, and explains why the cooling branch exhibits longer time constants in the identified models.

The clear separation of time scales ($\tau^{\prime }<\tau$) supports the chosen cascade architecture: a faster inner loop can regulate module face temperature while the slower outer loop controls the chamber air temperature. The small delays (~1 min) are non-negligible relative to the thermal time constants and benefit from explicit compensation (Smith predictor) when aggressive tracking is required. The strong gain asymmetry between heating and cooling motivated the design choice to implement separate inner controllers $C_{D}^{h}\left(s\right)$ and $C_{D}^{c}\left(s\right)$ (see Section 2.5).

### 3.2. Controller-Tuning Results

#### 3.2.1. Candidate Tunings and Comparative Metrics

Initial controller settings are listed in Table 6 for the outer controller $C_{T}\left(s\right)$ and the two inner controllers, $C_{D}^{h}\left(s\right)$ and $C_{D}^{c}\left(s\right)$, computed from the model-based tuning methods (ZN, CC, CHR, AMIGO, SIMC, MDPM). Table 7 reports the error indicators (ISE, IAE, ITAE, TV) obtained from the initial tuning comparison.

The most relevant outcome is that SIMC produced the best overall compromise across the error metrics (lowest IAE, ITAE; competitive ISE, TV), while some other methods (e.g., CHR) produced low actuator activity (TV) but substantially worse tracking. MDPM generated aggressive gains that yielded high TV.

#### 3.2.2. Final Tuning and Experimental Refinement

SIMC candidates were used as the starting point for hardware fine-tuning. Table 8 lists the final tuned parameters. These settings reflect modest changes from the initial SIMC values, which provided good baseline performance, and only small adjustments were required to compensate for modelling mismatch and measurement noise.

**Figure 9:**
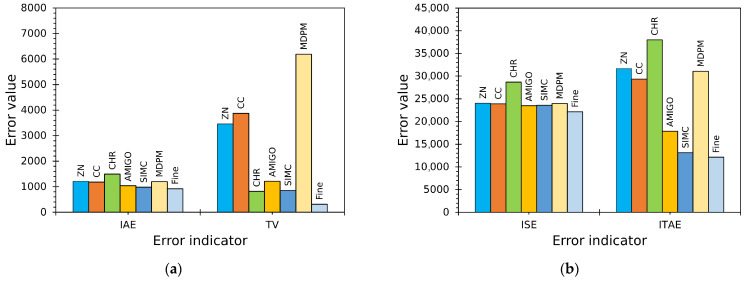
Comparison of performance indicators for final tuned controllers: (**a**) IAE and TV; (**b**) ISE and ITAE.

A graphical comparison of performance indicators is shown in Figure 9. The figure highlights that the fine-tuned controllers achieve the lowest IAE, ISE, ITAE, and TV at moderate levels—indicating improved tracking with minimal actuator effort. Fine-tuning led to a significant reduction in both ITAE and TV compared to most model-based tuning rules, indicating faster settling with lower long-term error and the lowest actuator effort.

#### 3.2.3. Practical Performance and Robustness

The following key points were observed:

- Setpoint tracking: with the final tuning, the cascade controller tracked step references and multi-segment profiles with small steady-state error and acceptable overshoot. The Smith predictor and 2-DOF structure were important to reduce delay-induced degradation during relatively fast transients.
- Switching between heating and cooling: the use of two inner controllers and a 0.5 °C hysteresis band prevented chattering at sign changes; bumpless transfer logic avoided actuator jumps at switch instants.
- Actuator effort and accuracy: fine-tuning reduced both tracking errors and actuator effort, achieving low duty-cycle activity without sacrificing accuracy. For practical deployments where energy consumption is critical, the SIMC-derived tuning with minor refinements provided the best overall balance.
- Robustness: final parameters were tested under variations in ambient temperature (from 5 to 25 °C), thermal load, and with several repeated runs; no instability or controller saturation issues were observed.

### 3.3. Test Results with Real Refrigerated-Truck Temperature Profiles

Two 36-day validation experiments were conducted to evaluate the chamber’s ability to reproduce realistic refrigerated-truck temperature profiles under closed-loop control and to quantify long-term tracking performance across multiple thermal transients. Each run followed a time-varying reference trajectory synthesized from real temperature traces recorded inside refrigerated trucks using an intelligent temperature tracker [62]. The ambient laboratory temperature during the test was ~20 °C. Both validation tests were conducted with thermal load inside the chamber, consisting of approximately 4 kg of cherry tomatoes.

A separate 24-h demonstration compares the unmodified (ZN-tuned) PID and the final optimized PID (with 2-DOF, anti-windup, Smith predictor, and hysteresis) on the same reference profile to illustrate practical improvements in transient behaviour and steady error.

#### 3.3.1. Tracking Performance

**Figure 10:**
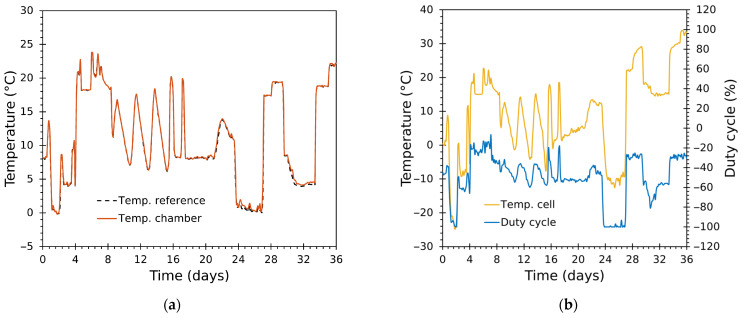
Test 1: full 36-day time series. (**a**) Reference and chamber ($T_{in}$) temperatures; (**b**) module temperature ($T_{cell}$) and duty cycle (D).

**Figure 11:**
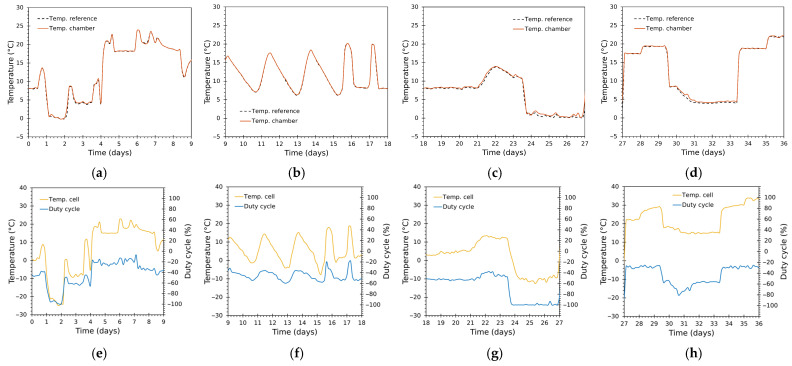
Test 1: zoom panels for a detailed view. (**a**–**d**) Reference and chamber ($T_{in}$) temperatures; (**e**–**h**) module temperature ($T_{cell}$) and duty cycle (D).

**Figure 12:**
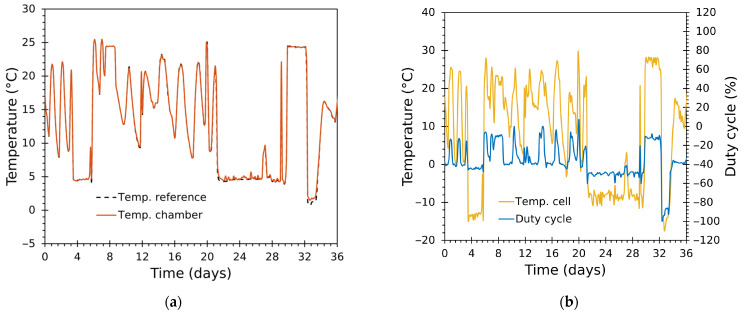
Test 2: full 36-day time series. (**a**) Reference and chamber ($T_{in}$) temperatures; (**b**) module temperature ($T_{cell}$) and duty cycle (D).

**Figure 13:**
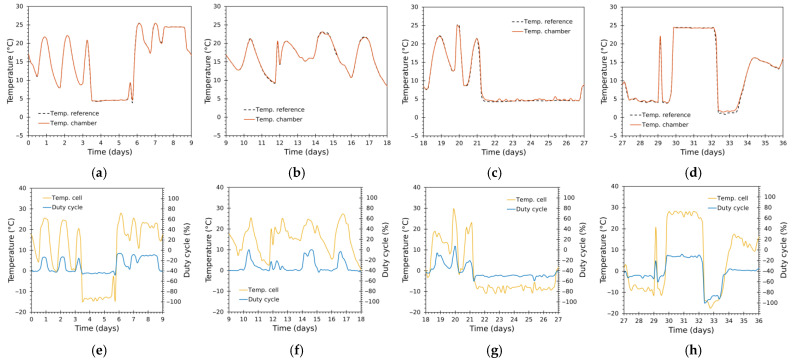
Test 2: zoom panels for a detailed view. (**a**–**d**) Reference and chamber ($T_{in}$) temperatures; (**e**–**h**) module temperature ($T_{cell}$) and duty cycle (D).

The system reproduced complex, multi-modal temperature trajectories with high fidelity throughout both 36-day runs, exercising the chamber across a wide range of temperatures and frequent transitions (Figure 10, Figure 11, Figure 12 and Figure 13). Figure 10 and Figure 12 show the complete thermal tests, while Figure 11 and Figure 13 depict zoomed details.

- The chamber air temperature tracked the reference closely (Figure 10a and Figure 12a). Global results show almost coincident traces for large fractions of the experiments covering a wide variety of operational regimes—prolonged holds, rapid ramps, frequent short transients.
- Zoomed windows (Figure 11a–d and Figure 13a–d) reveal the chamber’s behaviour during rapid transitions. Settling times following aggressive ramps were short relative to the typical time scales of transport events (orders of minutes to an hour), with minimal overshoot in most transitions.
- Even during very aggressive ramps or transients, settling was achieved with little or no overshoot, a direct consequence of: (i) the cascade control architecture; (ii) separate tuning for heating/cooling inner loops; and (iii) the Smith predictor alleviating delay effects.
- The inner-module temperatures, determined by the duty cycle in the cascade control, achieved larger amplitude excursions than the chamber air (Figure 10b, Figure 11e–h, Figure 12b and Figure 13e–h), as expected from system-identification results (Section 3.1). The duty cycle was optimal and responded rapidly during transitions thanks to the properly tuned controllers, avoiding unnecessary saturation and bringing the cells to their ideal operating point to achieve the desired temperature inside the chamber, while minimizing the module effort.
- Cumulative duty-cycle worked as an energy proxy: better tracking for extremely low values (near 0 °C) required higher duty activity. These sections show relatively larger deviations. For application contexts where energy is constrained, a slightly less aggressive tuning (increasing tolerance or applying explicit cost in tuning) would reduce energy at the cost of larger, but still acceptable, errors.
- The control system used higher duty bursts only when required, avoided prolonged saturation, and switched heating/cooling branches smoothly—the 0.5 °C hysteresis and bumpless transfer prevented chattering. The Smith predictor reduced the effective impact of transport delays and improved transient damping, while anti-windup prevented integrator accumulation during saturation.

#### 3.3.2. Regression and Residual Analysis of Chamber Temperature Tracking

**Figure 14:**
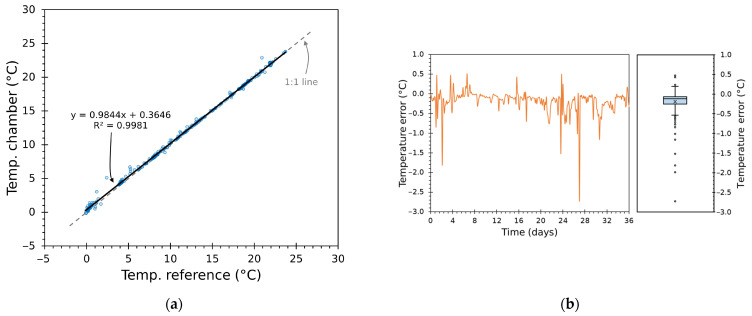
Test 1: (**a**) scatter plot of $T_{in}$ versus reference with identity line and linear regression ($R^{2}=0.9981$); (**b**) residual diagnostics with time series and boxplot of temperature error.

**Figure 15:**
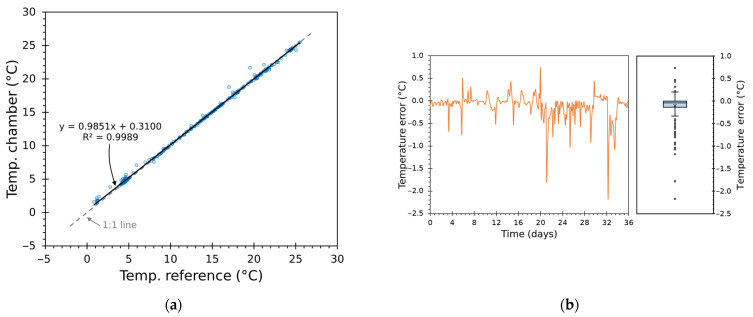
Test 2: (**a**) scatter plot of $T_{in}$ versus reference with identity line and linear regression ($R^{2}=0.9989$); (**b**) residual diagnostics with time series and boxplot of temperature error.

Scatter plots of chamber versus reference temperatures (Figure 14a and Figure 15a) yield highly linear behaviour. Linear regression lines overlap closely with the identity line—slopes very close to 1, negligible intercepts, and R^2^ of 0.9981 and 0.9989—confirming that the mapping was nearly perfect over large periods.

Residual analysis using time series and boxplots (Figure 14b and Figure 15b) highlights a tight, roughly symmetric error distribution centred around a small negative bias—indicating the system tends to sit slightly colder on average—with the majority of errors within ±0.5 °C. These results demonstrate consistent performance and quantify how closely the chamber followed the target trajectories.

Greater deviations are concentrated around rapid transition instants or low-temperature segments. The thermal inertia of the modules plus heatsink assembly limits how fast the air node can reach the new setpoint despite aggressive duty bursts; the Smith predictor and cascaded inner loop reduce but cannot entirely eliminate the finite transport delay and thermal mass. This behaviour is visible in Figure 10, Figure 11, Figure 12 and Figure 13 and is consistent with the statistical shape of the residual distributions.

From an experimental design standpoint, the results are robust: identical controller logic and tuning strategy produced very similar, high-quality tracking across two independently recorded truck traces, demonstrating reproducibility. Overall, the visual and regression diagnostics demonstrate that the chamber provides faithful, repeatable reproduction of long cold-chain histories.

#### 3.3.3. Thermal Behaviour of Peltier Cells and Chamber Air Temperature

**Figure 16:**
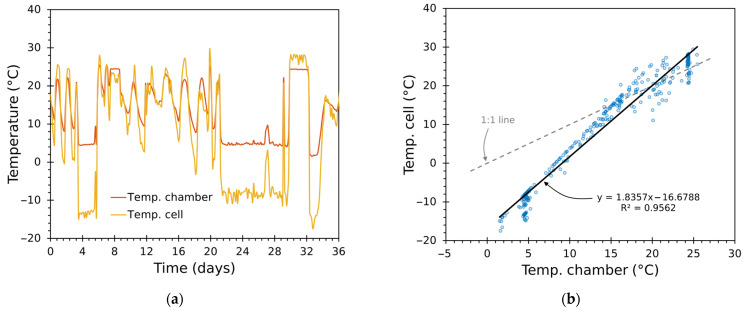
Test 1: thermal response of $T_{in}$ and $T_{cell}$, showing (**a**) time series and (**b**) scatter plot with identity line and linear regression.

**Figure 17:**
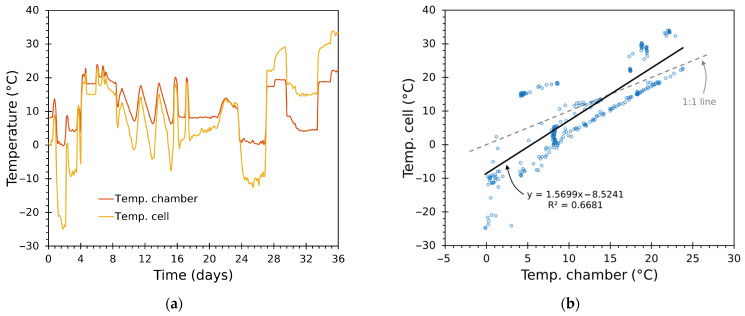
Test 2: thermal response of $T_{in}$ and $T_{cell}$, showing (**a**) time series and (**b**) scatter plot with identity line and linear regression.

Figure 16 and Figure 17 compare module-face temperature ($T_{cell}$) and chamber-air temperature ($T_{in}$) for the trials. Figure 16a and Figure 17a include a time series showing both temperatures together, while Figure 16b and Figure 17b depict a scatter plot with identity and regression lines.

The regression slopes exceed unity (around 1.84 and 1.57), indicating that module faces swing with larger amplitude than the air node: 1 °C of chamber air change corresponds to roughly 1.6–1.8 °C change on the Peltier face in these experiments. This is a direct consequence of the thermal coupling—module small thermal mass and direct, concentrated heat—and the convective resistance between cell faces and air.

Practically, the steeper slope implies the inner loop must tolerate larger module excursions—without saturating or causing excessive thermal stress—while the outer loop experiences a smoother, lower-amplitude target. The cascade structure compensates for this separation of magnitudes.

Moreover, because the mapping from module-face to chamber-air temperatures is approximately linear and repeatable, the use of the cascade design was appropriate: the inner loop stabilizes the fast, high-amplitude actuator dynamics, and the outer loop focuses on the smoother air temperature. Larger deviations between these magnitudes occur at lower and near-ambient temperatures in the tests.

In addition, the system demonstrated robust thermal control under variable humidity conditions encountered during the long-duration tests. Periodic condensation on cold surfaces was effectively managed by the chamber’s purge and drainage system, with no disruption to closed-loop operation. Short humidity transients caused minor, localized increases in temperature ripple, but tracking performance remained stable and accurate. These results support the chamber’s suitability for experiments involving realistic moisture dynamics. However, fully coupled temperature-humidity control would require dedicated humidification/dehumidification hardware and reconfiguration of the control architecture.

#### 3.3.4. Statistical Analysis and Evaluation Metrics

Table 9 reports the tracking metrics for the two 36-day trials, including individual and mean values. Both runs demonstrate consistently low errors and excellent agreement between chamber air temperature and the reference trace:

- Mean absolute error (MAE) was 0.1903 °C, while average mean squared error and its square root (MSE and RMSE) values were 0.1089 °C^2^ and 0.3282 °C, respectively.
- Median absolute errors (MedAE) were particularly small (0.1305 and 0.0700 °C), indicating that the bulk of the run was tracked with sub-tenth-to-few-tenths degree precision. MedAE being lower than MAE indicates a slightly skewed error distribution where a minority of larger deviations increases the mean, consistent with rare transients after aggressive ramps.
- Biases were slightly negative in both trials (mean −0.1471 °C), showing a small systematic tendency for the chamber to finish slightly colder than the reference on average; these small negative values are consistent with the rest of the results.
- The low standard deviation (SD) of the residuals (average 0.2921 °C) ensures that experiments requiring repeatable thermal histories will not be dominated by uncontrolled temperature variability.
- Regression fits between chamber and reference temperatures returned extremely high coefficients of determination (average $R^{2}=0.9985$), confirming near-linear, near-ideal tracking over very large periods.

Evaluation metrics were calculated as presented in Equations (27)–(33):

(27) $$\mathrm{MAE}=\frac{1}{N}\sum_{k=1}^{N}\left|T_{in,k}^{ref}-T_{in,k}\right|$$

(28) $$\mathrm{MedAE}=\mathrm{median}\left\{\left|T_{in,1}^{ref}-T_{in,1}\right|,\;\ldots ,\;\left|T_{in,N}^{ref}-T_{in,N}\right|\right\}$$

(29) $$\mathrm{MSE}=\frac{1}{N}\sum_{k=1}^{N}{\left(T_{in,k}^{ref}-T_{in,k}\right)}^{2}$$

(30) $$\mathrm{RMSE}=\sqrt{\mathrm{MSE}}=\sqrt{\frac{1}{N}\sum_{k=1}^{N}{\left(T_{in,k}^{ref}-T_{in,k}\right)}^{2}}$$

(31) $$\mathrm{Bias}=\frac{1}{N}\sum_{k=1}^{N}\left(T_{in,k}^{ref}-T_{in,k}\right)$$

(32) $$\mathrm{SD}=\sqrt{\frac{1}{N-1}\sum_{k=1}^{N}{\left[\left(T_{in,k}^{ref}-T_{in,k}\right)-\mathrm{Bias}\right]}^{2}}$$

(33) $$R^{2}=1-\frac{\sum_{k=1}^{N}{\left(T_{in,k}^{ref}-T_{in,k}\right)}^{2}}{\sum_{k=1}^{N}{\left(T_{in,k}^{ref}-{\bar{T}}_{in}^{ref}\right)}^{2}}$$

where $T_{in,k}^{ref}$ is the reference temperature, $T_{in,k}$ the measured temperature, N the sample size, and ${\bar{T}}_{in}^{ref}=\frac{1}{N}\sum_{k=1}^{N}T_{in,k}^{ref}$.

#### 3.3.5. Controller Improvements Comparison

**Figure 18:**
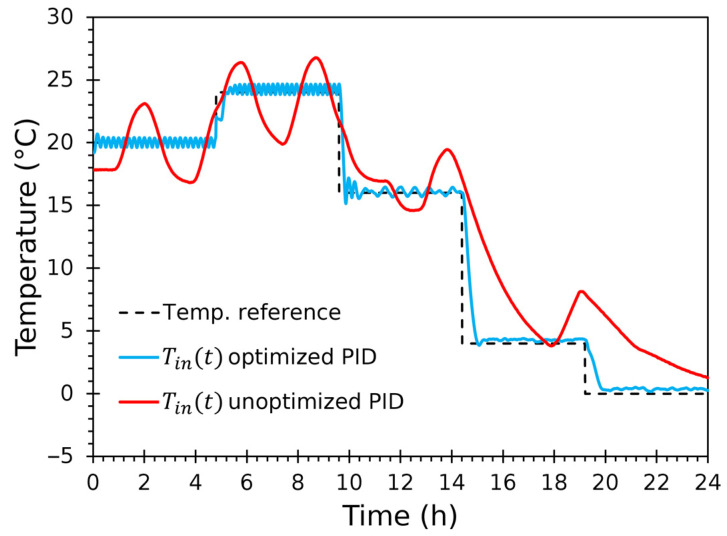
PID performance comparison under a 24-h test, showing unoptimized (ZN-tuned) PID and final optimized PID (2-DOF, anti-windup, Smith predictor, and hysteresis) on the same reference profile.

**Figure 19:**
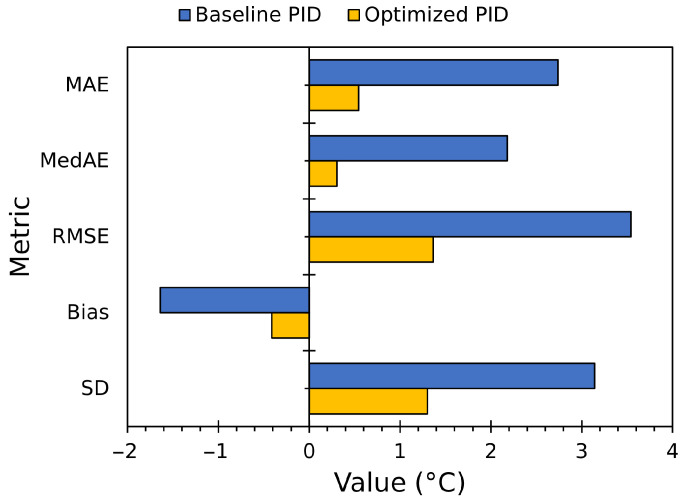
Evaluation metrics for baseline PID versus final optimized PID, for the 24-h test.

The 24-h controller comparison (Figure 18 and Figure 19; Table 10) shows the practical impact of the control improvements. The time-series plot shows that the baseline controller produced persistent overshoot and large steady-state offsets, while the optimized control tracked setpoints with substantially smaller ripple and faster recovery, avoiding overshoot.

Baseline PID produced large steady and transient errors (MAE≅2.74 °C, RMSE≅3.54 °C, $R^{2}=0.8971$) and clear over/under-shooting, while optimized PID reduced MAE roughly fivefold to 0.54 °C, cut RMSE by more than half to 1.37 °C, and raised $R^{2}$ to 0.9804. Median absolute error fell from about 2.18 °C to 0.30 °C, demonstrating that the optimized controller maintains reference tracking, eliminating large, frequent deviations and greatly improving both steady-state and transient fidelity.

Transient steps were tracked with short settling times and minimal overshoot under the optimized controller. The Smith predictor and cascade arrangement were decisive in mitigating dead-time effects and preventing integrator windup during prolonged actuator saturation. The inner loop’s separate tuning for heating and cooling compensated for the thermoelectric asymmetry observed during identification and reduced chattering when the polarity was switched. Also, the 0.5 °C hysteresis plus bumpless transfer produced smooth transitions in the duty cycle and avoided sharp actuator steps.

In this short, rapid trial, it is easier to observe that the temperature ripple about the reference increased near ambient (~20 °C) and diminished at lower setpoints (Figure 18). This behaviour matched physical expectations: near ambient, the control authority of the modules interacted with very low net thermal gradients (walls conducted roughly at ambient), making fine adjustments more sensitive and increasing the relative contribution of convection to ripple. At low temperatures, the net heat flow was dominated by the cells, and the larger ΔT produced a more stable control action, since larger heat losses allowed for higher temperature smoothing.

Closed-loop control was robust, and the hysteresis-based switching between heating and cooling inner controllers avoided chattering during near-zero crossing segments. The validation tests demonstrate that the proposed hardware and control approach reproducibly emulate realistic cold-chain temperature histories with high accuracy over multi-week durations. The optimized controller delivers a dramatic improvement over a naive PID implementation, producing tracking quality suitable for controlled physical-twin experiments and for generating training datasets for digital-twin models.

The results demonstrate that the combination of cascade architecture, 2-DOF weighting, Smith predictor, anti-windup, and hysteresis is highly effective for thermoelectric actuation in the presence of thermal inertia and polarity switching. The 2-DOF structure decouples setpoint transients from disturbance rejection, allowing aggressive disturbance rejection without large setpoint overshoot. Anti-windup prevents integrator accumulation during saturation to ensure that recovery from long heating/cooling branches is much faster, and the Smith predictor neutralizes the effect of dead-times.

Finally, separating inner-loop controllers for heating and cooling matches the asymmetry in thermoelectric dynamics and avoids the suboptimal compromise of a single controller. In addition, the optimized controller also improved actuator behaviour: the duty-cycle traces (Figure 10b and Figure 12b) show that control action is concentrated into timely bursts rather than continuous saturation, reducing average actuator stress and likely improving energy efficiency and component longevity.

Recent studies have explored advanced control strategies for thermoelectric systems, including model predictive control [27,31], fuzzy logic [29,30], and adaptive sliding-mode approaches [25]. While these methods can offer strong performance in specific scenarios, they often require high computational resources or complex modelling. In contrast, our approach combines cascade PID control with modern enhancements (2-DOF, anti-windup, Smith predictor, hysteresis), achieving high accuracy with low computational overhead, making it suitable for embedded and low-cost platforms.

## 4. Conclusions

This paper presents a compact, portable climate chamber based on Peltier modules together with an identification-driven control strategy that reliably reproduces realistic cold-chain temperature histories. The primary novelty lies in the control design: a cascaded architecture combining two-degree-of-freedom PID controllers, Smith predictor’s dead-time compensation, anti-windup back-calculation, and hysteresis-based bumpless switching, plus separate inner-loop tuning for heating and cooling to address the asymmetric thermoelectric behaviour of Peltier cells. This practical combination delivers fast, stable tracking across frequent polarity changes and aggressive thermal transients while remaining computationally light and suitable for embedded hardware.

Experimentally, the system shows strong real-world performance. Two independent 36-day validation tests achieved consistently low long-run errors (MAE≅0.19 °C, RMSE≅0.33 °C, $R^{2}=0.9985$), and a focused 24-h comparison demonstrated that the optimized controller improves performance, reducing settling times and overshoot. These results confirm that the chamber can serve as a reproducible physical twin for multi-week cold-chain experiments, producing high-quality, timestamped thermal traces that are suitable for training predictive shelf-life models and seeding digital-twin workflows.

Sensors embedded within the climate chamber play a critical role in achieving accurate and responsive thermal regulation. By providing high-resolution, real-time measurements of air temperature, module-face temperature, and relative humidity, they enable the control system to precisely replicate the dynamic thermal conditions experienced during refrigerated transport. This fidelity is essential to the chamber’s function as a physical twin, enabling reproducible experiments and generating reliable data to train predictive models. In turn, these models support decision-making in the cold chain, helping to anticipate spoilage and optimize logistics, thereby contributing to the reduction in perishable food waste.

Moreover, this work discusses an important operational trade-off: achieving faster transient response typically requires higher module currents that reduce instantaneous COP, while energy-efficient operation needs modest currents and small face ΔT. Recognizing this trade-off enables flexible operation modes (speed-optimized vs. energy-optimized) and points to practical extensions such as COP-aware control, multi-stage Peltier cells, or fan-speed modulation to tune convective coupling. The prototype also proved robust to realistic humidity transients: condensate was handled without interrupting closed-loop control, though precise humidity control would require additional actuators and retuning.

Our contribution combines a compact, reversible actuation platform with a tailored, robust control approach, high-fidelity sensor instrumentation, and extensive long-duration validation. The result is a practical, low-complexity tool for reproducible cold-chain emulation that lowers the barrier for experimental studies of perishable-food quality and supports the development of data-driven supply-chain optimizations aimed at reducing food waste.

## Figures and Tables

**Table 1:** Peltier cell extracted parameter values (TEC1-12708).

Peltier Cell Parameter	Value
$V_{max}$	15.4 V
$I_{max}$	8.5 A
$\Delta T_{max}$	66 K
$T_{h0}$	298 K
R	1.41 Ω
K	0.772 W/K
S	0.0517 V/K

**Table 2:** Operating point values used for system linearization.

Operating Point	Heat Case	Cool Case
$D_{0}$	50%	−50%
$I_{cell_{0}}$	2.37 A	2.92 A
$T_{cell_{0}}$	344.46 K	256.52 K
$T_{in_{0}}$	335.32 K	263.00 K

**Table 3:** Tuning methods for PID parameters.

Method	$K_{p}$	$T_{i}$	$T_{d}$
Ziegler–Nichols	$1.2\frac{T}{\kappa \theta }$	2θ	0.5θ
Cohen–Coon	$\frac{T}{\kappa \theta }\left(\frac{4}{3}+\frac{\theta }{4T}\right)$	$\frac{32+6\theta /T}{13+8\theta /T}\theta$	$\frac{4}{11+2\theta /T}\theta$
Chien–Hrones–Reswick	$0.6\frac{T}{\kappa \theta }$	T	0.5θ
AMIGO	$\frac{1}{\kappa }\left(0.2+0.45\frac{T}{\theta }\right)$	$\frac{0.4\theta +0.8T}{\theta +0.1T}\theta$	$\frac{0.5T\theta }{0.3\theta +T}$
SIMC	$\frac{2T+\theta }{4\theta \kappa }$	$\min\left(T+\frac{\theta }{2},8\theta \right)$	$\frac{T\theta }{2T+\theta }$

**Table 4:** Multiple Dominant Pole Method for 2-DOF PID tuning.

Parameter	Value
$s_{*}$	$-\frac{3}{\theta }-\frac{1}{2T}+\sqrt{\frac{3}{\theta^{2}}+\frac{1}{4T^{2}}}$
$K_{p}$	$\frac{1}{\kappa }\left[\theta^{2}Ts_{*}^{3}+\theta \left(3T+\theta \right)s_{*}^{2}+\theta s_{*}-1\right]e^{\theta s_{*}}$
$T_{i}$	$-2\cdot \frac{\theta^{2}Ts_{*}^{3}+\theta \left(3T+\theta \right)s_{*}^{2}+\theta s_{*}-1}{\theta s_{*}^{3}\left(\theta Ts_{*}+2T+\theta \right)}$
$T_{d}$	$-\frac{1}{2}\cdot \frac{\theta^{2}Ts_{*}^{2}+\theta \left(4T+\theta \right)s_{*}+2\theta +2T}{\theta^{2}Ts_{*}^{3}+\theta \left(3T+\theta \right)s_{*}^{2}+\theta s_{*}-1}$
λ	$\min\left\{\frac{2}{T_{i}\left|s_{*}\right|},\;1\right\}$
μ	$\min\left\{\frac{1}{T_{i}T_{d}s_{*}^{2}},\;1\right\}$

**Table 5:** Identified values of transfer-function constants from step tests.

Parameter	Operating Mode	Value
Γ	-	0.5441
τ	-	26.85 min
L	-	1.17 min
Ω	Heating	144.93 °C
	Cooling	34.67 °C
$\tau^{\prime }$	Heating	8.84 min
	Cooling	18.17 min
$L^{\prime }$	Heating	0.83 min
	Cooling	1.00 min

**Table 6:** Initial PID settings for $C_{T}\left(s\right)$, $C_{D}^{h}\left(s\right)$ and $C_{D}^{c}\left(s\right)$ derived from model-based tuning methods.

Method	Parameter	$C_{T}\left(s\right)$	$C_{D}^{h}\left(s\right)$	$C_{D}^{c}\left(s\right)$
ZN	$K_{p}$	50.61	0.09	0.63
	$T_{i}$	2.34	1.66	2.00
	$T_{d}$	0.59	0.42	0.50
	λ	0.70	0.70	0.70
	μ	0.00	0.00	0.00
CC	$K_{p}$	56.69	0.10	0.71
	$T_{i}$	2.83	1.97	2.41
	$T_{d}$	0.42	0.29	0.36
	λ	0.70	0.70	0.70
	μ	0.00	0.00	0.00
CHR	$K_{p}$	25.31	0.04	0.31
	$T_{i}$	26.85	8.84	18.17
	$T_{d}$	0.59	0.42	0.50
	λ	0.70	0.70	0.70
	μ	0.00	0.00	0.00
AMIGO	$K_{p}$	19.35	0.03	0.24
	$T_{i}$	6.66	3.59	5.30
	$T_{d}$	0.58	0.41	0.49
	λ	0.70	0.70	0.70
	μ	0.00	0.00	0.00
SIMC	$K_{p}$	21.55	0.04	0.27
	$T_{i}$	9.36	6.64	8.00
	$T_{d}$	0.57	0.39	0.48
	λ	0.70	0.70	0.70
	μ	0.00	0.00	0.00
MDPM	$K_{p}$	32.72	0.06	0.41
	$T_{i}$	4.08	2.68	3.42
	$T_{d}$	0.30	0.21	0.26
	λ	0.45	0.47	0.45
	μ	0.66	0.70	0.67
All methods	η	0.05	0.05	0.05
	$K_{b}$	0.90	1.32	1.06
	ν	0.50	0.50	0.50

**Table 7:** Performance comparison of initial controller tunings based on standard error and control effort indicators.

Method	ISE	IAE	ITAE	TV
ZN	24,041.48	1215.30	31,680.38	3462.21
CC	23,895.09	1184.06	29,363.43	3875.00
CHR	28,697.20	1492.31	37,997.72	820.81
AMIGO	23,484.53	1040.11	17,875.05	1213.74
SIMC	23,583.20	979.87	13,136.75	851.85
MDPM	23,983.11	1200.79	31,059.62	6190.95

**Table 8:** Final tuned controller parameters and performance indicators.

	PID								Performance Indicators			
	$K_{p}$	$T_{i}$	$T_{d}$	λ	μ	η	$K_{b}$	ν	ISE	IAE	ITAE	TV
$C_{T}\left(s\right)$	21.97	8.02	0.44	0.38	0.00	0.06	0.60	0.55				
$C_{D}^{h}\left(s\right)$	0.05	6.81	0.36	0.73	0.09	0.04	1.30	0.55	22,148.04	921.33	12,128.27	314.52
$C_{D}^{c}\left(s\right)$	0.26	7.22	0.53	0.69	0.17	0.06	0.96	0.58				

**Table 9:** Summary of tracking performance metrics for the two 36-day validation tests.

Metric	Test 1	Test 2	Mean
MAE	0.2217 °C	0.1588 °C	0.1903 °C
MedAE	0.1305 °C	0.0700 °C	0.1002 °C
MSE	0.1315 °C^2^	0.0864 °C^2^	0.1089 °C^2^
RMSE	0.3626 °C	0.2939 °C	0.3282 °C
Bias	−0.1918 °C	−0.1024 °C	−0.1471 °C
SD	0.3082 °C	0.2759 °C	0.2921 °C
$R^{2}$	0.9981	0.9989	0.9985

**Table 10:** Performance comparison between baseline (ZN-tuned) and optimized PID controllers over a 24-h test.

Metric	Baseline PID	Optimized PID
MAE	2.7377 °C	0.5438 °C
MedAE	2.1808 °C	0.3047 °C
RMSE	3.5425 °C	1.3659 °C
Bias	−1.6395 °C	−0.4111 °C
SD	3.1425 °C	1.3030 °C
$R^{2}$	0.8971	0.9804

## Data Availability

The data presented in this study are available on request from the corresponding author.

## Abbreviations

The following abbreviations are used in this manuscript:

**Acronyms**	
2-DOF PID	Two-Degree-of-Freedom PID Controller
ABS	Acrylonitrile Butadiene Styrene
ADC	Analogue-to-Digital Converter
AMIGO	Approximate M-constrained Integral Gain Optimization
CC	Cohen–Coon
CHR	Chien–Hrones–Reswick
COP	Coefficient of Performance
DC	Direct Current
EPS	Expanded Polystyrene
IAE	Integral Absolute Error
ISE	Integral Square Error
ITAE	Integral of Time-multiplied Absolute Error
MAE	Mean Absolute Error
MDPM	Multiple Dominant Pole Method
MedAE	Median Absolute Error
MPC	Model Predictive Control
MSE	Mean Squared Error
NTC	Negative Temperature Coefficient
PID	Proportional–Integral–Derivative controller
PLA	Polylactic Acid
PWM	Pulse Width Modulation
RMSE	Root Mean Square Error
SD	Standard Deviation
SIMC	Skogestad Internal Model Control
TV	Total Variation
ZN	Ziegler–Nichols
**Symbols**	
A	Internal surface area of the chamber ($m^{2}$)
$C_{D}$, $C_{D}^{c}$, $C_{D}^{h}$	Inner-loop PID controllers: general, cooling branch, heating branch
$C_{p}$	Specific heat of air ($J\cdot kg^{-1}\cdot K^{-1}$)
$C_{p_{c}}$	Specific heat of the module assembly ($J\cdot kg^{-1}\cdot K^{-1}$)
$C_{T}$	Outer-loop PID controller for chamber air temperature
$COP_{c}$, $COP_{h}$	Coefficient of performance for cooling and heating
D	Duty cycle of the PWM control signal
$D_{0}$	Duty cycle operating point
G	Geometrical factor (area/length) (m)
$G_{D}$	Transfer function from duty cycle to module temperature
$G_{T}$	Transfer function from module temperature to chamber air temperature
I	Electrical current (A)
$I_{cell}$	Current through the thermoelectric module (A)
$I_{{cell}_{0}}$	Current through the module operating point (A)
$I_{max}$	Maximum rated current of the module (A)
K	Thermal conductance of the module ($WK^{-1}$)
$K_{b}$	Anti-windup back-calculation constant
$K_{p}$	Proportional gain of the PID controller
$k_{\theta }$	Thermal conductivity ($Wm^{-1}K^{-1}$)
L, $L^{\prime }$	Transport delay (dead time) in the thermal systems (min)
$m_{c}$	Lumped mass of the module assembly (kg)
$N_{c}$	Number of thermoelectric modules
$N_{u}$	Number of thermocouple pairs in a module
$P_{e}$	Electrical power input to the module (W)
${\dot{Q}}_{c}$	Heat absorbed at the cold side of the module (W)
${\dot{q}}_{cell}$	Heat produced or absorbed by a single module (W)
${\dot{Q}}_{h}$	Heat rejected at the hot side of the module (W)
${\dot{Q}}_{Peltier}$	Ideal Peltier heat flow (W)
S	Seebeck coefficient ($VK^{-1}$)
$s_{\alpha }$	Elemental Seebeck coefficient ($VK^{-1}$)
T	Temperature (K or °C)
$T_{c}$	Cold face temperature of the module (K)
$T_{cell}$	Temperature of the module face (K)
$T_{{cell}_{0}}$	Module temperature operating point (K)
$T_{d}$	Derivative time of the PID controller (min)
$T_{h}$	Hot face temperature of the module (K)
$T_{h0}$	Reference module hot-side temperature from datasheet (K)
$T_{i}$	Integral time of the PID controller (min)
$T_{in}$	Chamber air temperature (K)
$T_{in_{0}}$	Chamber air temperature operating point (K)
$T_{\infty }$	External ambient temperature (K)
U	Overall heat-transfer coefficient of the chamber enclosure ($Wm^{-2}K^{-1}$)
V	Internal volume of the chamber ($m^{3}$)
$V_{CC}$	Supply voltage rail for the module (V)
$V_{cell}$	Voltage applied to the module (V)
$V_{e}$	Electrical voltage (V)
$V_{max}$	Maximum rated voltage of the module (V)
R	Electrical resistance of the module (Ω)
$R^{2}$	Coefficient of determination
$R_{\theta }$	Effective thermal resistance between modules and chamber air ($KW^{-1}$)
Γ	Gain of module-temperature to air-temperature transfer function (°C/°C)
ΔT	Temperature difference (K)
$\Delta T_{max}$	Maximum temperature difference across the module (K)
η	Derivative filter constant in the PID controller
λ, μ	Setpoint weighting factors in 2-DOF PID controller
ν	Low-pass filter constant in the Smith predictor
Π	Peltier coefficient (V)
ρ	Air density ($\mathrm{kg}\cdot m^{-3}$)
$\rho_{e}$	Electrical resistivity (Ωm)
τ, $\tau^{\prime }$	Time constants of the thermal systems (min)
Ω	Gain of duty-cycle to module-temperature transfer function (°C)
